# Assessment of Insulin-related Knowledge among Healthcare Professionals in a Large Teaching Hospital in the United Kingdom

**DOI:** 10.3390/pharmacy7010016

**Published:** 2019-01-30

**Authors:** Amie Bain, Sallianne Kavanagh, Sinead McCarthy, Zaheer-Ud-Din Babar

**Affiliations:** 1 School of Applied Sciences, University of Huddersfield, Huddersfield HD1 3DH, UK; sineadmcc12@hotmail.co.uk (S.M.); z.babar@hud.ac.uk (Z.-U.-D.B.); 2 Sheffield Teaching Hospitals NHS Foundation Trust, Sheffield S5 7AU, UK; Sallianne.Kavanagh@sth.nhs.uk

**Keywords:** diabetes mellitus, medication error, insulin, secondary care, prescribing, hospital, education

## Abstract

Despite numerous strategies introduced to promote the safe use of insulin, insulin-related medication errors persist. Our aim was to examine the knowledge and self-reported confidence of a range of healthcare professionals regarding insulin use in a large teaching hospital in the North of England. A 16-item electronic questionnaire was prepared in light of locally reported insulin-related incidents and distributed electronically to all healthcare professionals at the hospital over a 4-week study period. A range of healthcare professionals, including nurses, pharmacists, pharmacy technicians, junior doctors and consultants, completed the questionnaires (n = 109). Pharmacists achieved the greatest percentage of mean correct answers overall (49%), followed by consultant doctors (38%) and pharmacy technicians (37%), junior doctors (34%) and nurses (32%). Healthcare professionals were mainly “slightly confident” in their knowledge and use of insulin. Confidence level positively correlated to performance, but number of years’ experience did not result in higher confidence or performance. This small-scale study allowed for a broad assessment of insulin-related topics that have been identified both nationally and locally as particularly problematic. Identifying knowledge gaps may help tailor strategies to help improve insulin knowledge and patient safety.

## 1. Introduction

Insulin is a high-risk medication used in the treatment of both type 1 and type 2 diabetes mellitus [1]. Inappropriate use of insulin may result in hyper- or hypoglycaemia, potentially causing avoidable patient harm or even death [2,3]. The need to ensure that patients receive the appropriate insulin therapy is therefore crucial to prevent serious complications [4,5,6].

Insulin remains one of the top medicines implicated in adverse drug events, as well as being involved in medication errors worldwide [7]. In the United Kingdom, a report by the National Patient Safety Agency (NPSA) in 2010 highlighted the number of incidents where insulin contributed to patient harm or death and outlined immediate actions for organisations as a result [3]. Despite such initiatives, insulin errors remain a problem [8], with evidence from the 2017 National Diabetes Inpatient Audit suggesting that almost half of patients treated with insulin experience a medication error related to their insulin (49%), an increase compared to previous years (46% in 2016) [9].

Insufficient knowledge regarding the safe use of insulin among healthcare professionals may contribute to medication errors and patient harm [10,11]. The ever-increasing number of insulin products available, as well as the complexities associated with its use, may contribute to a lack of confidence and competence among healthcare professionals when they prescribe and administer insulin to patients [12]. Furthermore, in recent years, new groups of healthcare professionals are now involved in documenting, prescribing and administrating insulin therapy. For example, pharmacy technicians are frequently undertaking medicines reconciliation processes on admission to hospital [13], and healthcare assistants and carers often administer insulin to patients in the community [14,15]. This highlights the need to offer and tailor insulin education to an expanding variety of professional groups in order to encourage the safe use of insulin.

Previous studies have reported low confidence and gaps in nurses’ knowledge with respect to insulin use, with only around 50% of participants correctly answering questions on aspects of insulin administration [16,17,18]. Doctors’ results appear to be consistent with this [16]. Other studies have shown that pharmacists achieve higher overall scores (86%, compared with 57% for doctors and 50% for nurses), with some inter-professional variation between scores for questions regarding insulin characteristics and prescription compared with preparation and administration [19].

It has been suggested that a lack of knowledge on insulin prescribing, dispensing, dosing and administration can contribute to medication errors [20], and that introducing insulin-related educational interventions can decrease error by nurses and doctors [21,22,23]. Overall, the literature indicates that there is a general lack of insulin-related knowledge amongst healthcare professionals [16,17,21,22,24,25]. Further exploration is therefore required to improve patient safety. The scarcity of published literature with regards to numerous healthcare professionals managing insulin therapy in hospital provided the rationale for this study.

The aim was to measure and examine staff knowledge and self-reported confidence of a range of healthcare professionals regarding insulin products and administration in hospitals. The objectives of the study included:

- To identify specific knowledge gaps amongst healthcare professionals regarding insulin products and dosing regimens.
- To determine correlations between insulin knowledge, self-reported confidence level, professional group and years of experience of healthcare professionals.
- To describe healthcare professionals’ experiences of previous involvement in insulin-related medication errors, and their recommendations for improving insulin safety.

## 2. Materials and Methods 

The study was conducted at a large teaching hospital in the North of England between December 2016 and January 2017. The hospital is part of one of the largest and busiest NHS hospital trusts in England, providing services to 2.3 million patients across five hospitals and 40 community sites. An electronic questionnaire was prepared by two pharmacists and a pharmacy student in light of previously reported insulin-related incidents at both a national and local level (Figure S1, supplementary information). Answers to questions were agreed by consensus and reflected both national and local guidance regarding the appropriate and safe use of commonly used insulin products. 

Validity and content of the questionnaire were examined by piloting the questionnaire with a convenience sample of 10 academic and clinical pharmacists. Several iterations addressed any reported ambiguities and a refined version was agreed by consensus of the study team. A specialist panel from the hospital’s clinical effectiveness unit also reviewed and approved the questionnaire and study procedure. Ethical approval was not required as per the hospital’s criteria for research studies.

The questionnaire consisted of 16 items, including both multiple-choice and open questions, in order to allow achievement of the study objectives. All qualified and registered healthcare professionals practising at the hospital who were involved in the care of patients were invited to complete the questionnaire, irrespective of profession or number of years of experience (n = approx. 6000). Due to the range of healthcare professionals involved in the prescribing and administration of medicines (e.g., including non-medical prescribers from a range of backgrounds), broad inclusion criteria was thought to better reflect current practice.

The hospital communications team distributed an email to all staff employed at the hospital in January 2017, inviting those involved in patient care to complete the online questionnaire. The email included a link to allow staff to directly access and self-complete the questionnaire via their web browser, and was resent after 2 weeks to increase visibility and participation. The online questionnaire remained live for 4 weeks, after which it was closed, and the results analysed. Informed voluntary consent was implied by completion of the online anonymous questionnaire. Participants were requested not to refer to any information resources and to provide answers on the basis of their knowledge. There was no set time limit for completion of the questionnaire; participants were advised that it should take around 5 min to complete. 

Participants were asked to disclose their professional group, number of years in practice, their clinical area, and confidence level regarding insulin products and their use. Due to the size of the organisation (which employs over 17,000 clinical and non-clinical staff), such information was not thought to compromise the anonymity of the data obtained.

### Data Analysis Approach

Data was generated and inputted directly and electronically via the online questionnaire platform and translated into a Microsoft Excel 2016 document for descriptive analysis. Any questions that were answered incorrectly, as “I don’t know” or left unanswered, were considered incorrect. Questions that required selection of more than one option to comprise a correct answer were only considered correct if all correct options were chosen. Answers to open-ended questions were inductively thematically analysed by a single researcher. Findings were confirmed by an independent pharmacist to increase rigour.

## 3. Results

A total of 113 questionnaires were completed. The total number of clinical staff who were available to access and answer the questionnaire during the study period was unknown due to the nature of central electronic distribution. Out of those who responded, 4 participants were either pre-qualification or not actively involved in direct patient care (e.g., pre-registration pharmacy trainees, biomedical scientists) and were therefore excluded from the final analysis. 

Respondents included in the final analysis (n = 109) comprised of 36 nurses (33%), 33 hospital pharmacists of all grades (30%), 20 doctors (19%)—of whom 16 (15%) were junior doctors (foundation doctors and registrars) and 4 (4%) were consultants—, 18 pharmacy technicians (17%), 1 dietician (1%) and 1 operational department practitioner (1%). All 109 participants included in the final analysis completed the questionnaire, allowing analysis of all participant answers. 

A range of clinical specialities were represented in the study, including surgery (24%), cardiology (18%), diabetes and endocrine, acute medicine, haematology and care of the elderly, (8% each), respiratory, critical care, renal (6% each) and psychiatry and head and neck (4% each).

### 3.1. Confidence Level and Insulin-Related Knowledge

**Figure 1:**
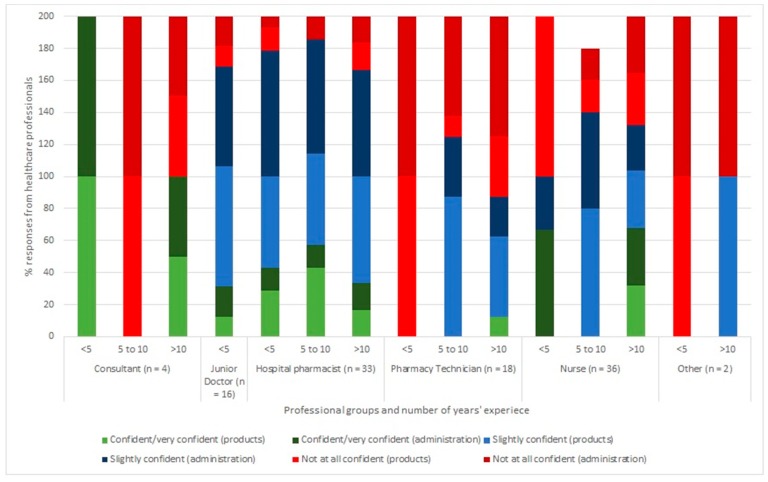
Percentage of healthcare professionals’ self-reporting levels of confidence regarding their knowledge of insulin products (%) and administration (%). Results are grouped according to profession and number of years’ experience.

Most professionals reported being “slightly confident” in their knowledge of insulin products and regimens; only 21% of respondents stated that they were confident, or very confident in their knowledge of insulin. Figure 1 shows the self-reported confidence level of respondents from different professional groups regarding their knowledge of insulin products and regimens. Pharmacy technicians and “other” professional groups reported the least confidence in their knowledge compared with pharmacists, nurses and doctors. Overall confidence levels did not tend to increase with number of years’ experience.

Table 1 shows that respondents who felt “very confident” on their knowledge of insulin products and regimens scored the highest overall mean marks for the questionnaire. Those who felt “confident” about their knowledge of insulin products scored higher for those questions compared to those with other confidence levels. Those who felt “very confident” about their knowledge of insulin regimens scored higher for those questions compared to those with other confidence levels. 

### 3.2. Professional Group, Experience and Insulin-Related Knowledge

**Figure 2:**
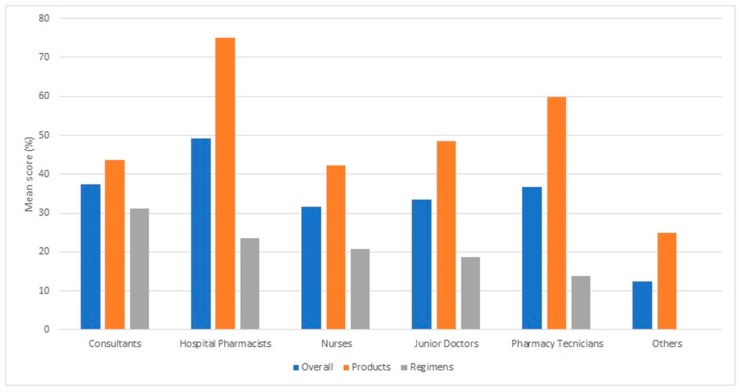
Mean percentage correct answers for each professional group according to question type, and overall.

The mean score overall for all professional groups answering all questions was 38%. All respondents answered the questions involving insulin products better than the questions involving insulin regimens (with overall mean scores of 56% and 20% being achieved, respectively). Mean percentages of correct answers returned for each professional group are illustrated in Figure 2.

Pharmacists achieved the greatest percentage of mean correct answers overall (49%), followed by consultant doctors (38%), pharmacy technicians (37%), junior doctors (34%), nurses (32%) and others (13%). Pharmacists returned the most correct answers with respect to insulin products (75%), followed by pharmacy technicians (60%), with doctors and nurses scoring similarly for these questions (42–48%). Consultants returned the most correct answers with respect to insulin regimens (31%), followed by pharmacists, junior doctors, and nurses, who scored similarly for these questions (19–23%). The responses for each individual question are shown in Table 2.

Questions concerning long-acting insulin (Q9 and 10) were answered very well by most groups. Questions regarding duration of action (Q11 and 12) were answered poorly by all groups; the question returning the least amount of answers (Q6) concerned administration of insulin before meals. The other mealtime administration question (Q7) was answered more successfully by all participants.

Doctors and nurses showed greater awareness of the duration of rapid acting insulin (Q12) than their pharmacy and nursing colleagues, whereas pharmacy staff demonstrated greater knowledge regarding the duration of action of mixed insulin (Q11). Pharmacy staff and consultants were more aware of products that contained non-standard concentrations of insulin (Q13) than their junior doctor and nurse colleagues. Junior doctors showed the greatest variety in performance between questions amongst the professional groups.

Overall, the number of years’ experience had only a very slight impact on performance. Interestingly, those with less than 5 years’ experience had the greatest mean score (40%) compared to those with between 5 and 10 years of experience (38%) and more than 10 years’ experience (36%).

### 3.3. Insulin Incidents and Interventions

Twenty-nine (27%) professionals reported being involved in, or identifying, a previous insulin prescription, administration or management error, or near-miss. Most responses (45%) described incidents involving the wrong form of insulin being prescribed (e.g., Humalog instead of Humalog Mix 25), others described insulin being prescribed at the wrong time(s) of day, inappropriate omission of insulin (prescription or administration) and erroneous use of intravenous insulin and concomitant fluids.

Seventy-two (66%) participants suggested measures to improve insulin safety in inpatients, most of which (58%) stated a need for more education on this topic, explaining that more regular training and education sessions, study days and regular e-learning modules could help increase their knowledge and help them to deal with patients with diabetes better. Seventeen people (24%) suggested increasing the number and use of available resources to improve insulin safety, for example, quick-reference guides and increased specialist input. Seven people (10%) suggested tighter regulation with respect to insulin (e.g., limit formularies, better segregation of storage and second checking for administration). Other suggestions included allowing patients to self-administer where capable, prescribers having their mistakes highlighted so they may reflect and improve their practice, and better communication between care providers.

## 4. Discussion

This study allowed for a broad assessment of insulin-related topics that have been identified both nationally and locally as particularly problematic. It is the first study of its kind to include the range of professional groups that are involved in insulin prescribing and administration processes in hospital, for example pharmacy technicians, who routinely gather and present important insulin information to their medical, pharmacy and nursing colleagues.

### 4.1. Confidence Level and Insulin-Related Knowledge

We report a general lack of insulin-related knowledge and confidence across all professions, despite interventions to improve insulin safety in recent years. This echoes the findings of Derr et al., who report that only a minority of the nurses and doctors included in their study felt very comfortable managing diabetes [16]. We found a greater variation in overall scores for different confidence levels, however, this could be due to the questionnaire addressing specific insulin topics, rather than diabetes in general. A slight correlation was found between knowledge and self-reported confidence, although this did not appear to be significant.

### 4.2. Professional Group, Experience and Insulin-Related Knowledge

This study found that pharmacists achieved the greatest mean score amongst the professional groups, although certain topics seemed to be understood by different professions. For example, more nurses returned correct answers regarding insulin regimens than their junior medical colleagues. These results support those of Lee et al. [19] and may be expected, as pharmacists are trained specifically in the knowledge of medication use, and nurses routinely administer insulin to patients. Our results thus support previous recommendations that pharmacists may be utilised to help minimise the amount of insulin-related medication errors [26].

We also found that apart from pharmacists, pharmacy technicians demonstrated a greater level of knowledge regarding insulin products compared with other professions, which is a previously unreported result. Our findings would, therefore, also support pharmacy technician involvement in relevant insulin-related medication gathering and technical screening processes in hospital.

Both nurses and junior doctors did not score highly regarding non-standard insulin concentrations in this study. This supports Segal’s findings [27], which identified that some professionals were unable to identify the different strengths of insulin products, and a lack of knowledge on this contributed to errors. Recent national alerts have highlighted this problem, and healthcare professionals are encouraged to become familiar with these products in order to avoid future errors and patient harm [28,29]. Results from this study support targeted interventions to junior medical and nursing staff regarding the prescription and administration of non-standard insulin concentrations (e.g., >100 units/mL). Topics that were poorly understood by all professions (e.g., insulin administration times and duration of action) may also be incorporated into more general educational strategies and interventions to improve insulin safety.

The number of years worked did not have a significant impact on performance in this study, and supports the findings of Derr et al. [16] who specifically examined nurses and doctors’ performance. Further studies would be required in order to explore how and what experience(s) may correlate to greater insulin-related knowledge. Information regarding grade bandings and specialities (e.g., diabetes specialist nurses) of healthcare professionals involved, as well as prescriber status, may also be helpful in this respect.

### 4.3. Insulin Incidents and Interventions

Staff reported previous incidents involving inappropriate omission of insulin, which was previously highlighted by the National Patient Safety Agency [3] and a previous study conducted by Sharpe et al. [18]. Most respondents cited education and resource availability as important interventions to improve insulin safety. Other studies describe how educational strategies can improve staff knowledge and confidence and reduce insulin errors, supporting the introduction of more intense and targeted educational approaches [23,30].

### 4.4. Limitations of the Study

Although the questionnaire was assured for content validity, further benefit may have been achieved with multi-professional input in the initial stages. Convenience sampling, although respectful of busy front-line staff pressures, may have introduced bias, and the response rate was limited; however, the study was conducted in a typical large teaching hospital in England. The setting would not be predicted to be very different from most other hospital settings, and therefore the study findings could be applied in the broader context.

## 5. Conclusions

This study demonstrates significant knowledge gaps around insulin products and regimens across a greater variety of healthcare professions than has been reported previously. Combined with a low level of self-reported confidence with respect to insulin knowledge, this highlights the need for further interventions to tackle insulin-related medication errors in hospitals. Healthcare professionals’ opinions regarding improving insulin safety are reported, and support further educational and resource-based interventions. Results highlight the relative strength of pharmacy professionals’ knowledge in this area, demonstrating their value in supporting the safe use of insulin in hospitals.

## Figures and Tables

**Table 1:** Self-reported confidence level and mean percentage correct answers returned.

	Very Confident	Confident	Sligtly Confident	Not at All Confident
**Number of respondants (N)**				
**Insulin products questions**	3	20	58	28
**Insulin regimens questions**	5	17	51	36
**Mean (%) correct answers**				
**Insulin products questions (overall)**	67 (58)	75 (54)	57 (39)	38 (22)
**Insulin regimens questions (overall)**	50 (58)	24 (43)	25 (44)	8 (25)

Note: N = numbers of respondents (healthcare professionals) selecting level of confidence. Numbers in parentheses represent % correct answers overall for comparison.

**Table 2:** Percentages of respondents from each professional group returning correct answers for each knowledge-based question.

Multiple-choice Question Asked (Answers in Italic)	Pharmacist (n = 33)	Pharmacy Technician (n = 18)	Junior Doctor (n = 16)	Consultant (n = 4)	Nurse (n = 36)	Other (n = 2)	Overall (n = 109)
**6. Which insulin should be administered 15 min prior to meals?** *Humulin M3, Humulin S*	15	17	13	25	3	0	**11**
**7. Which insulin(s) are to be administered at mealtimes?** *Humalog*	64	33	25	50	33	0	**41**
**8. Which insulin preparation should never be given at night?** *Humalog Mix 50, Apidra, NovoRapid, Humulin S*	3	0	13	0	19	0	**9**
**9. Levemir (detemir) is** *a long-acting insulin*	91	67	81	50	50	50	**70**
**10. Which of the following is a basal (long-acting) insulin?** *Lantus (glargine)*	97	83	100	75	83	50	**89**
**11.What is the duration of action for NovoMix 30?** *16–24 h*	33	33	0	0	6	0	**17**
**12.Subcutaneous (SC) Actrapid should not be administered at intervals less than** *4 h*	12	6	25	50	28	0	**19**
**13.Which of the following insulin products contain 100 units/mL?** *Humulin S, Lantus, Novorapid*	79	56	13	50	31	0	**47**
**Average**	49	37	34	38	32	13	**38**
**Max/Min**	97/3	83/0	100/0	75/0	83/3	50/0	**89/9**

## Supplementary Materials

The following are available online at http://www.mdpi.com/2226-4787/7/1/16/s1, Figure S1: insulin questionnaire.
