# Supplementary material for: Assessment of Insulin-related Knowledge among Healthcare Professionals in a Large Teaching Hospital in the United Kingdom
Source: Pharmacy (Basel). 2019 Jan 30;7(1):16. doi: 10.3390/pharmacy7010016 (PMC6473239; doi:10.3390/pharmacy7010016)
Supplement: Supplementary file 1 [file pharmacy-07-00016-s001.pdf]

1. What is your profession?
a) Hospital pharmacist
b) Junior Doctor
c) Consultant
d) Pharmacy technician
e) Nurse
f) Other (please state):

2. How many years have you worked in this profession?

3. In what clinical area are you currently working?

4. How confident are you about your knowledge of insulin products?
a) Very confident
b) Confident
c) Slightly confident
d) Not at all confident

5. How confident are you about your knowledge of insulin dosage regimens?
a) Very confident
b) Confident
c) Slightly confident
d) Not at all confident

6. Which of the following insulins is recommended to be administered 15 minutes prior to meals? (Select one or more answers)
a) Humulin M3
b) NovoRapid
c) Humalog
d) Humulin S
e) I don't know

7. Which of the following insulins are to be administered at mealtimes? (Select one or more answers)
a) Levemir
b) Humulin S
c) Humalog
d) Humulin M3
e) I don't know

8. Which insulin preparation should never be given at night? (Select one or more answers)
a) Humalog Mix50
b) Apidra
c) NovoRapid
d) Humulin S
e) I don't know

9. Levemir (detemir) is:
a) A rapid acting insulin
b) An intermediate-acting insulin
c) A long-acting insulin
d) A short-acting insulin
e) I don't know

10. Which of the following is a basal (long-acting) insulin? (Select one or more answers)
a) Apidra
b) NovoMix 30
c) Actrapid
d) Lantus (glargine)
e) I don't know

11. What is the duration of action for NovoMix 30?
a) 30 minutes
b) 3-5 hours
c) 6-8 hours
d) 16-24 hours
e) I don't know

12. Subcutaneous (SC) Actrapid should not be administered at intervals less than:
a) 4 hours
b) 3 hours
c) 2 hours
d) 1 hour
e) I don't know

13. Which of the following insulin products contain 100units/mL? (Select one or more answers)
a) Humulin S
b) Lantus
c) Toujeo
d) NovoRapid
e) I don't know

14. Have you ever identified, or been involved in, an insulin prescription, administration or management error/near miss?
a) Yes
b) No

15. If so, please give details.

16. What would you suggest needs to be done to improve insulin safety in hospitals?

Figure S1. Insulin questionnaire distributed to healthcare professionals. Correct answers are highlighted.
